# Using Behavior Change Theory to Understand How to Support Screening for Traumatic Brain Injuries Among Women Who Have Experienced Intimate Partner Violence

**DOI:** 10.1089/whr.2020.0097

**Published:** 2021-08-16

**Authors:** Blake Nicol, Paul van Donkelaar, Karen Mason, Heather Gainforth

**Affiliations:** ^1^ School of Health and Exercise Sciences, University of British Columbia Okanagan, Okanagan, Canada.

**Keywords:** behavior change theory, intimate partner violence, Theoretical Domains Framework, traumatic brain injury

## Abstract

***Background:*** Women who experience intimate partner violence (IPV) are at a high risk for traumatic brain injuries (TBIs). Women's shelters may be an ideal location for TBI screening. Behavior change theory can help understand factors that influence screening at women's shelters and develop interventions to promote screening.

***Objective:*** To use behavior change theory to understand the local context of women's shelters, factors influencing screening for TBIs among staff who work at women's shelters, and co-develop intervention recommendations to promote screening of TBIs at women's shelters.

***Methods:*** The research was conducted in three phases in partnership with the Kelowna Women's Shelter. In phase 1, participants (staff at women's shelters across Canada) completed an online survey that assessed their current TBI screening behaviors, knowledge of TBIs, and factors influencing screening. In phase 2, participants (staff at women's shelters in the Okanagan) completed an interview regarding the factors that influence screening for TBIs. In both phases, factors were analyzed using the Theoretical Domains Framework. In phase 3, intervention recommendations were co-developed using the Behavior Change Wheel.

***Results:*** In phase 1, findings indicate that participants (*n* = 150) lack skills (mean = 2.1, standard deviation [SD] = 1.9) and knowledge (mean = 2.9, SD = 2.2) with regard to screening and are nervous to screen (mean = 3.0, SD = 2.4) for TBIs. In phase 2, 194 barriers to screening for TBI were extracted from 10 interviews with staff members. Prominent domains included knowledge (37%), beliefs about capabilities (16%), and environmental context and resources (15%). Finally, in phase 3, five intervention recommendations were co-developed for interventions aiming to promote TBI screening in women's shelters.

***Conclusions:*** This thesis was the first theory-based study to develop intervention recommendations for promoting screening of TBIs at women's shelters. The recommendations have the potential to increase TBI screening at women's shelters ultimately improving the quality of life of women who have experienced a TBI from IPV.

## Background

Women who experience intimate partner violence (IPV) are at high risk of traumatic brain injuries (TBIs). TBIs are a pathophysiological disruption in brain functioning caused by sudden impact or acceleration deceleration trauma of the head.^1^ The head, neck, and face are most likely to be injured during IPV, which can often result in a TBI.^2–8^ A review found that 30%–74% of victims of IPV suffered a TBI as a result.^9^ TBIs have the potential to affect many aspects of one's life and could make it especially difficult for women who have experienced a TBI from IPV. Screening women survivors of IPV for TBIs may contribute to decisions about how best to provide necessary supports to help them with TBI recovery, leaving abusive relationships, and avoiding future violence, abuse, and neglect. To the best of our knowledge, previous research has not examined the prevalence of TBI screening within women's shelters. Screening for TBIs at women's shelters may help increase referrals to TBI supports and improve the quality of life of women who have experienced a TBI from IPV.

Previous research has explored potential TBI screening techniques and tools for IPV. It has been proposed that screening methods should (1) include prompts relevant to the events that can result in TBI in this population; (2) allow for safe and private endorsement of an event; and (3) offer ease of administration by IPV knowledgeable staff without the need for special training in TBI.^10^

Furthermore, research found nine screening tools, and five that may work within the context of IPV, including the HELPS tool.^10^ Although there have been no validated tools for detecting IPV-related TBI, the HELPS tool has been adapted for use in an IPV context,^11^ and the HELPS screening tool is the best available screening tool and should be considered an initial screen for all women known to have experienced abuse^12^; however, many shelters are not using TBI screening tools as part of the services they provide.

Currently, there are issues to consider with screening IPV survivors for TBI. Screening tools have yet to be validated for this population. Most validated TBI tools are used on young healthy adults who experience a “one-time” injury, and studies suggest that women are more vulnerable to TBI and have worse functional outcomes post-TBI.^12^ To improve support for women who have experienced IPV, interventions are needed to ensure staff at women's shelters screen for TBIs.

Behavior change theory can be used to support the systematic development of evidence-based interventions. Behavior change theory is valuable because it can explain why, when, and how a behavior does or does not occur, and why an intervention succeeds or fails. It can also help improve the replicability of interventions.^13–15^

The Behavior Change Wheel (BCW) provides a comprehensive and systematic process for developing behavior change interventions.^16^ From inward out, the BCW consists of the COM-B (Capability, Opportunity, Motivation, and Behavior) model, intervention functions, and policy categories. The COM-B model states that behavior is in an interactive system involving capability, opportunity, and motivation and interventions need to change one or more of them to change behavior.^16^

A COM-B analysis and behavioral diagnosis can be performed to help understand what components of the COM-B (Capability, Opportunity, and Motivation) model need to be targeted in an intervention to promote screening of TBIs at women's shelters. Subsequently, the outer layers of the BCW can be used systematically to identify intervention options in terms of intervention functions (*i.e.*, broad categories of means by which an intervention can change behavior) and policy categories (*i.e.*, ways in which an intervention could be implemented).

While the COM-B model provides an understanding of components influencing behavior, several studies have used the Theoretical Domains Framework (TDF) to understand barriers and facilitators to behavior change. Contributors to the development of the TDF included health psychology theorists, health service researchers, and health psychologists.

One hundred twenty-eight explanatory constructs drawing on 33 psychological theories were identified with regard to changing behavior. In 2012, the TDF was validated and a second current version was developed, which consists of 14 theoretical domains (knowledge; skills; social/professional role and identity; beliefs about capabilities; optimism; beliefs about consequences; reinforcement; intentions; goals; memory, attention, and decision processes; environmental context and resources; social influences; emotion; and behavioral regulation).^17^

A combination of the BCW and the TDF can help better understand the barriers that staff at women's shelters face in screening their clients for TBIs and how to best provide interventions to target these barriers. The APEASE (Affordability, Practicability, Effectiveness and cost-effectiveness, Acceptability, Side effects/safety, and Equity) criteria can then be used to further narrow down relevant intervention options.

Due to the high prevalence of TBI in women who experience IPV and the lack of TBI screening among this population, the overarching aim of this study was to use the BCW and TDF to understand and improve screening for TBIs among women who have experienced IPV by women's shelter staff. The specific aims of this study were to (1) understand if staff at women's shelters screen for TBIs, (2) use behavior change theory to understand barriers to screening for TBIs among shelter staff, and (3) co-develop intervention recommendations with regard to screening women for TBIs at women's shelters.

## Methods

### Design

Aligned with the aims, this study involved three phases. Phase 1 was a cross-sectional survey using a questionnaire to ask staff from women's shelters across Canada about their current practices, knowledge of TBIs, the factors influencing whether they screen for TBIs, and demographic details. In phase 2, interviews were conducted to better understand the local context of women's shelters in one geographic area and to provide a more detailed understanding of the factors influencing whether staff screen for TBIs. Finally, in phase 3, the research team used the BCW to identify relevant intervention functions and policy categories. The APEASE criteria were then used to co-develop intervention recommendations for promoting screening of TBIs at women's shelters. The study protocol was approved by the UBCO Behavioural Research Ethics Board (H17-00143) and all participants provided informed consent prior to taking part.

### Research context and approach

This study used a pragmatic and integrated knowledge translation (iKT) approach by partnering with a women's shelter. Our team consisted of researchers and the executive director of a women's shelter. The executive director of the women's shelter was involved in all steps of the research process and helped develop the research question, the survey, the interview guide, and assisted with analyzing data, and disseminating findings. The research consisted of a mix-methods approach using surveys and interviews to best address the research aims.

### Phase 1: cross-sectional survey

#### Participants and recruitment

Participants included staff members from organizations across Canada who support women who have experienced IPV. Staff members were recruited onsite at three IPV conferences held in Canada. Members from the research team attended the conferences to recruit participants for an online- or paper-based survey. A $100 gift card prize draw was offered as an incentive for participants. The survey was offered in both English and French.

#### Design

The survey consisted of measures to assess staff's (1) current knowledge of TBIs, (2) current practices with regard to screening for TBIs at their organization, (3) perceptions of the theoretical domains that influence screening for TBIs at their organization, and (4) demographics.

##### Knowledge of TBIs

Participants' current knowledge regarding screening for TBIs at their organization was assessed using an 18-item questionnaire adapted from Kurowski et al.^18^ Finally, participants were also asked “How common do you think traumatic brain injury is within your client population?” and were provided with the following response options: <10%, 11%–25%, 26%–50%, 51%–80%, and >80%.

##### Current practices

Participants' current practices with regard to screening for TBIs at their organization were assessed using one yes or no item. Specifically, participants were asked “Whether it's your organization's practice or not, have you ever screened for traumatic brain injuries in your work?”

##### Factors influencing screening for TBIs

The factors that influence participants' screening for TBIs were assessed using a 13-item questionnaire developed using the TDF, which was adapted by selecting statements from a TDF questionnaire for use in implementation research,^19^ and tailoring the items to an IPV-TBI context. To further probe any additional factors that may have influenced screening behaviors, participants were also asked “What would stop you from screening your clients for traumatic brain injuries?”

#### Analysis

In phase 1, general descriptives were reported for staff's current practices, knowledge of TBIs, theoretical domains that influence screening for TBIs, and demographics. Responses to open-ended questions were independently and deductively coded into TDF domains by two coders. Interrater agreement on the coding into TDF domains was calculated using Cohen's kappa^20^ and prevalence-adjusted bias-adjusted kappa (PABAK).^21^

Six steps of thematic analysis outlined by Braun and Clarke^22^ were conducted to determine themes among the TDF domains that were extracted from the open-ended question regarding factors that influence screening. Two other members of the research team served as critical sources of feedback on the themes throughout the analysis process. Key domains to target in an intervention were identified through an evidence-based matrix to produce the behavioral diagnosis.^14^

### Phase 2: interviews

#### Participants and recruitment

Participants were staff members from women's shelters. Participants were recruited through partnership with an Executive Director of a women's shelter. Staff members were sent an email with details of the study and were offered a $20 gift card incentive to participate in the interview.

#### Design

Interviews were ∼30 minutes long, using a semi-structured interview guide that was informed by Atkins et al.^23^ The objectives of phase 2 were to understand (1) the local context of women's shelters and (2) the factors that influence staff at women's shelters with regard to screening for TBIs. By taking a qualitative approach, this phase aimed to provide further insight into the findings of phase 1.

#### Analysis

In phase 2, general descriptives were reported for staff demographics. The audio recordings were transcribed verbatim, and two coders independently extracted the barriers and facilitators with regard to the factors that influence staff screening for TBIs. The coders met after every two transcripts to compare barriers and facilitators extracted and resolve any disagreements through a discussion. Where consensus could not be reached, a third expert coder was consulted. Intercoder agreement was measured using kappa and PABAK. A thematic analysis was also conducted in phase 2 to determine themes among barriers extracted from interviews. Key domains to target in an intervention were identified through an evidence-based matrix to produce the behavioral diagnosis.^14^

### Phase 3: policy recommendations

#### Design and analysis

The research team met to discuss the behavioral diagnoses developed from phase 1 and phase 2. Using evidence-based matrices,^14^ intervention functions and policy categories within the BCW that were linked to the theoretical factors influencing screening behaviors were identified. These data were presented to the research team, who then brainstormed intervention recommendations. Team members applied the APEASE (Affordability, Practicability, Effectiveness and cost-effectiveness, Acceptability, Side effects/safety, and Equity) criteria to determine appropriate intervention functions and policy categories and to co-develop intervention recommendations.^14^

## Results

### Phase 1

#### Participants

One hundred fifty-two people participated in the survey in 2018 from three different conferences; however, response rates varied depending on the question. The most common education level was a bachelor's degree (32.4%), followed by a master's degree (24.5%) and a college degree (22.3%). Most participants were white (73.7%), followed by Aboriginal (First Nations, Metis, Inuk) (11.7%).

#### Current practices and knowledge of TBIs

Three-quarters of participants had never formally screened for TBIs in their work (75.3%; *n* = 110). Staff scored an average of 12/18 (standard deviation [SD] = 1.99) on the knowledge questionnaire.

Staff were very good at identifying symptoms of TBI (*i.e.*, emotional changes [98.7% correct], sensitivity to noise and/or light [98.7 correct], nausea and/or vomiting [98.6 correct], difficulty in concentrating and/or remembering [99.3% correct], slower reaction time [99.3% correct], confusion [98.7% correct], and headache [98.6% correct]). However, they struggled to differentiate symptoms associated with TBI from symptoms not associated with TBI (*i.e.*, unusual thirst [41.3% correct], hallucinations [16.4% correct], shortness of breath [27.2% correct], and tingling in feet [25.3% correct]).

#### Results of TDF analysis to understand barriers and facilitators to screening for TBIs

The lowest rated domains were skills, knowledge, emotion, and memory, attention, and decision processes. The highest rated domains were intentions, beliefs about capabilities, social/professional role and identity, optimism, and beliefs about consequences (Table 1). The most frequent barriers extracted from the open-ended question “What would stop you from screening your clients for traumatic brain injuries?” were social influences (*n* = 73) and environmental context and resources (*n* = 17) (kappa = 0.94, PABAK = 0.98).

**Table 1.** Theoretical Domains Framework Domains Rated on a 7-Point Likert Scale (1 = Strongly Disagree, 7 = Strongly Agree) on Survey

Domain	Rating	Standard deviation
Skills	2.12	1.90
Knowledge	2.90	2.21
Emotion	2.97	2.36
Memory, attention, and decision processes	3.13	2.00
Environmental context and resources	3.51	2.35
Social influences	4.01	2.48
Intentions	4.26	2.53
Beliefs about capabilities	4.50	2.40
Social/professional role and identity	4.59	2.34
Optimism	4.65	2.54
Beliefs about consequences	6.13	1.75
Range	All domains had a range of scores from 1 to 7	

Subthemes emerged from the TDF domains of “social influences” and “environmental context and resources.” The subthemes related to the domain of social influences included: staff safety, client distress, lack of consent, and inability to screen. The subthemes related to the domains of environmental context and resources were time constraints, lack of staff, and lack of screening tool. Table 2 outlines the TDF domain and each subtheme within the domains. Quotes to support each subtheme and domain are provided.

**Table 2.** Theoretical Domains Framework Themes Extracted from the Survey Question “What Would Stop You from Screening for a Traumatic Brain Injury?” in Phase 1 Using Thematic Analysis

TDF domain theme	Subtheme	Exemplar quotes
Social Influences	Client Aggression and Staff Safety: Concerns related to the client being aggressive or unsafe towards staff.	“Client is showing aggression towards me and angry.”
	Causing Client Distress: Concerns that the client will feel distressed as a result of answering the screening questions.	“I would be fearful that it would contribute to further trauma for the person having to recollect the events of what happened.”
	Client Emotional State: Concerns that the client may be in crisis and may not be in an emotional state to respond to screening questions.	“If the client had very recently experienced trauma and was too upset to sit through the screening.”
	Lack of Consent: Client refusing to be screened by staff.	“If the client does not agree to the screening.”
	Inability to Screen: Concerns that the client may not have the psychological or physical capability to respond to the screening questions. Barriers cited related to disability status, use of substances, literacy, and so on.	“If the client was non-verbal, unable to hear, see etc.”
	No Reports of Injury to the Head: Clients reports that they have not sustained any injuries to the head.	“If they state that they have never been hit in the head.”
Environmental Context and Resources	Time Constraints: Concerns that they would not have enough time to complete the screening, and concerns that screening could take longer than described.	“Lack of time to do the screening at work.”
	Lack of Staff: Concerns that additional staff will be needed to implement screening while other duties are also still being performed.	“Being single staffed.”
	Lack of Screening Tool: Concerns that a screening protocol is not available.	“Not having a screening tool.”
Social/Professional Role and Identity: Concerns related to beliefs that it is someone else's role within or outside the organization (*e.g.*, medical professional) and/or screening is outside their scope of practice.	N/A	“A sense that I was not a medical professional, and this might be out of my realm of service.”
Knowledge: Concerns related to needed additional training and knowledge to understand TBIs and screening for TBIs.	N/A	“Lack of understanding.”
Beliefs about Capabilities: Concerns related to staff's confidence in their abilities to screen for TBIs.	N/A	“Not having data previous to injury to compare with.”
Skills: Concerns related to staff feeling that they have inadequate training to screen for TBIs.	N/A	“Lack of training.”
Goals: Related to staff prioritizing other tasks before screening for TBIs.	N/A	“Need medical attention ASAP.”
Beliefs about Consequences: Concerns with how screening of TBIs will benefit their clients.	N/A	“What is the benefit to our clients?”

TBIs, traumatic brain injuries; TDF, Theoretical Domains Framework.

The lowest rated barriers from the 7-point Likert scale consisted of skills, knowledge, emotion, and environmental context and resources. Furthermore, through the open-ended question, the domain of social influences was also a relevant barrier toward staff screening for TBIs. These findings suggest that the COM-B components, physical and psychological, physical and social opportunity, and automatic motivation, are factors that must change in order for staff to begin screening for TBIs at women's shelters. All nine intervention functions and all seven policy categories are relevant for targeting the above COM-B components.

### Phase 2

#### Participants

Participants (*n* = 10) were on average 45.3 (SD = 14.41) years old. They had an average of 10.83 (SD = 8.37) years of experience working with women who have experienced IPV and 4.93 (SD = 3.92) years of experience at their current organization. Most participants (70%) had at least a bachelor's degree, and the ethnicity of most was white (80%).

#### Results of TDF analysis to understand barriers and facilitators to screening for TBIs

In total, the coders extracted 194 barriers, which were categorized into 8 domains. The domains consisted of knowledge (*n* = 72), beliefs about capabilities (*n* = 32), environmental context and resources (*n* = 30), skills (*n* = 18), social influences (*n* = 13), social/professional role and identity (*n* = 12), beliefs about consequences (*n* = 9), and goals (*n* = 8). The interrater reliability for coding the TDF domains were between “substantial” and “almost perfect” (kappa = 0.76, PABAK = 0.94).

Subthemes emerged from the TDF domain environmental context and resources and social influences. Subthemes that emerged within the domain environmental context and resources included: lack of staff, lack of space, and lack of time. Whereas themes for the social influence domains include client's emotion, inability to screen, and building trust. Table 3 shows the TDF and subthemes with example quotes.

**Table 3.** Theoretical Domains Framework Themes Extracted from Interviews in Phase 2 Using Thematic Analysis

TDF domain theme	Subtheme	Exemplar quotes
Knowledge: Concerns related to needed additional training and knowledge to understand TBIs and screening for TBIs.	N/A	“I don't know very much. I would say probably on a scale of 1 to 10, I'd know 1.”
Beliefs about Capabilities: Concerns related to staff's confidence in their abilities to screen for TBIs.	N/A	“I would really like to feel more confident in myself moving ahead.”
Environmental Context and Resources: Concerns related to staff not being provided with what they need to screening for TBIs.	Lack of Staff: Concerns that additional staff will be needed to implement screening.	“We would need more staff working if you're going to have those.”
	Lack of Space: Concerns that they would not have enough space to screen for TBIs in their work.	“I mean, there is no space so it is a problem because we need more space.”
	Time Constraints: Concerns that they would not have enough time to complete the screening and concerns that screening could take longer than described.	“We can't give people more time.”
Social Influences: Concerns about how the client will react to screening for TBIs.	Alliance Between the Staff and Client: Concerns that clients may not trust staff enough to tell the truth or open-up during the screening process.	“Sometimes I just think there can be some hesitancy, especially because it's the first time that you're meeting this individual and they're coming and sharing really scary and really private details about their personal life that they may have never told people before. I just know that at times it can be difficult and there can be a lot of hesitancy for the individuals who are coming in and saying that information. So they're not always saying the full truth the very first time that you meet them. It might take a few more times of talking with them to get a better understanding of what they've experienced.”
	Causing Client Distress: Concerns that the client will feel distressed as a result of answering the screening questions.	“I don't want to open any wounds or any traumatic experiences for people.”
	Client Emotional State: Concerns that the client may be in crisis and may not be in an emotional state to respond to screening questions.	“The difficulty would be the women's emotional state. You know, if she's just come from being assaulted or if she's running from her partner.”
Skills: Concerns related to staff feeling that they have inadequate training to screen for TBIs.	N/A	“We haven't had any training through the shelter or anything that way in how to specifically deal with individuals who may be suffering from traumatic brain injuries.”
Social/Professional Role and Identity: Concerns related to beliefs that it is someone else's role within or outside the organization (*e.g.*, medical professional) and/or screening is outside of their scope of practice.	N/A	“I'm not a medical professional so I wouldn't say that I could assess them.”
Beliefs about Consequences: Concerns with how screening of TBIs will benefit their clients.	N/A	“Even if I did think that they had a brain injury it wouldn't change anything about the services they get or anything.”
Goals: Related to staff prioritizing other tasks before screening for TBIs.	N/A	“Basic needs need to be met first so if they have no money and no access to money and nowhere to live and nowhere to go and no job, those are sort of obviously the very top of the priority list for lots of people.”

The most prevalent barriers extracted from the interviews to screening for TBIs were knowledge (*n* = 72), beliefs about capabilities (*n* = 32), and environmental context and resources (*n* = 30). These findings suggest that the COM-B components, psychological capability, reflective motivation, and physical opportunity, are the factors that must change in order for staff to begin screening for TBIs. Again, all nine intervention functions and seven policy categories are relevant for targeting the above COM-B components.

### Phase 3

All nine intervention functions and all seven policy categories were identified as being relevant to promote screening of TBIs in women's shelters. From the APEASE meeting with the research team and the Executive Director of a women's shelter, six intervention functions and five policy categories were selected as being most likely to result in improved TBI screening in the context of women's shelters. For example, coercion was a relevant intervention function; however, during the APEASE meeting, coercion was determined to be not an acceptable intervention function for the population. Whereas the intervention function, training, met all APEASE criteria.

This process resulted in the co-development of five intervention recommendations (Table 4) to promote TBI screening at women's shelters. Table 4 includes relevant intervention functions and policy categories associated with each of the five intervention recommendations.

**Table 4.** Intervention Recommendations Developed from Behavioral Analysis of Phase 1 and Phase 2

Intervention recommendations	Description	Intervention functions	Policy categories
Establish formal policies and procedures requiring clients to be assessed for traumatic brain injuries.	These policies and procedures should be established by upper management and should make assessing for traumatic brain injuries part of the role of staff at women's shelter. It is important that staff understand that they are not diagnosing TBIs. The TBI assessment is to identify clients at risk for having experienced a TBI.	Persuasion Enablement	Regulations Guidelines
Training needs to be provided to staff who work in women's shelters.	This training should include education regarding knowledge of TBIs (*e.g.*, the CATT online tool, https://cattonline.com), real-world scenarios, and opportunities for practice and to receive feedback. Ideally, this training could be implemented using a train-the-trainer model that helps to establish “champions” in the workplace. If the train-the-trainer model is adopted, it is recommended that “champions” be someone in a long-term role due to what can be a high turnover rate of women's shelter staff.	Training Education Persuasion Modeling	Service Provision
Assess for traumatic brain injuries in a conversational style and not at intake.	A conversational assessment of TBIs should be done after intake once the client has settled into the women's shelter. This conversational assessment style should be done in a way that does not feel like a formal screening process.	Enablement	Service Provision
Educate clients about traumatic brain injuries.	Education provided to clients about TBIs should be basic information regarding some signs or symptoms of TBIs and information about TBI recovery. The information should be provided to the clients in a method that allows them to take the information with them (*e.g.*, pamphlets).	Enablement	Service Provision and Communication/Marketing
Develop a referral system for clients at risk for a traumatic brain injury.	Staff should refer clients at risk for a TBI through a referral system. The referral system should provide clients at risk for TBIs with the opportunity for a TBI diagnosis from a medical professional and TBI supports to help their recovery. If resources are too limited to develop a referral system, steps should be taken within the women's shelter to help with TBI recovery (*e.g.*, dim lights and provide quiet spaces).	Environmental Restructuring	Environmental and Social Planning

## Discussion

The aims of this study were to understand if, and how, women's shelters screen for TBIs, use behavior change theory to understand barriers and facilitators to screening for TBIs among shelter staff, and co-develop intervention recommendations for screening women for TBIs at women's shelters. Most staff (75.3%) at women's shelters have never formally screened for a TBI in their work. As a result, it is likely many women who experience a TBI from IPV go unidentified. An uptake of TBI screening by women's shelter staff could help women who have experienced IPV become aware of possible TBI. This could lead to possible TBI diagnosis by a medical professional and access to TBI-specific resources and supports.

Findings from our behavioral diagnoses indicate that knowledge is a necessary component to target in interventions that aim to ensure women are screened for TBIs at women's shelters. This is supported by another study that found the majority (84%) of support service providers reported no previous TBI training or education.^11^ Similar results were also found by Nemeth et al.^24^ A scoping review^11^ also found that a number of authors have emphasized the need for increase training for staff at women's shelters with regard to the identification of TBIs and its effects^9,25–31^; however, the lack of knowledge staff feel regarding screening for TBIs could also result from the misperception of what TBI screening involves.

It is important that staff understand they are not being asked to diagnose TBIs, but rather, through gaining important foundational knowledge, they can learn to recognize, respond to, and support women who are experiencing TBIs.

It is important to note, however, targeting knowledge is likely not enough to change TBI screening behaviors among staff at women's shelters. A common mistake when trying to change health-related behavior is the mindset that “knowledge and information drive behaviour.”^32^ Providing people with information and knowledge likely will not result in change, and this belief that mere knowledge can change behavior has the potential to undermine the effectiveness of interventions.^32^

Findings from the behavioral diagnoses indicated that changing TBI screening behavior in women's shelters is influenced by several factors. In addition to lacking knowledge, our findings showed that staff are likely not screening for TBIs because they are nervous, are influenced by their clients, and feel they lack the skills, resources, and confidence in their capabilities. Similar findings were reported by Haag et al.^11^ as all their participants reported feeling totally unprepared to only somewhat prepared to identify signs or symptoms of TBI.

Given the number of TDF domains relevant to screening behaviors, all nine intervention functions and all seven policy categories within the BCW were identified as being relevant to promote screening of TBIs in women's shelters. Therefore, a multicomponent intervention that targets intrapersonal, interpersonal, and systemic barriers should be developed.

Intervention recommendations were co-developed to support screening of TBIs in women's shelters for women who have experienced IPV. Recommendations were systematically developed using the TDF and the BCW. They highlight the need for development of policy and procedures with regard to screening for TBIs at women's shelters, training of staff at women's shelters, the importance of informal screening, the benefit of educating clients about TBI, and the benefit of having a referral network for women who have experienced a TBI from IPV. Each of the TDF domains influencing screening behaviors is discussed below, and alongside intervention recommendations that aim to target the domain.

Staff feel that they lack the skills and ability to screen for TBIs. This concern highlights the need to translate screening tools to real-world contexts. Similarly, Goldin et al. proposed that screening methods should offer ease of administration by IPV knowledgeable staff without the need for special training in TBI.^10^ Given our findings that staff are nervous to screen, have concerns about whether it is their role to screen, and want to maintain a strong bond with their client, it is likely screening tools need to be disseminated along with training that helps staff learn to use them both in a structured manner, and in an informal conversational style embedded in their daily practice.

To address this barrier, our intervention recommendations co-developed by the research team and the executive director of a women's shelter recommend providing training opportunities that allow staff to practice, and receive feedback, on delivering TBI-screening questions in both ways.

Lack of resources was a common barrier to screening for TBIs at women's shelters. Within this domain, four subthemes were found. Participants felt that they lacked the time, the appropriate number of staff, the space, and an appropriate tool to screen. In the survey, they were told screening would take “approximately 5 minutes.”

Staff may have felt that they lacked time because they believed they did not have an extra 5 minutes they could spare to screen for TBIs or because they believed they would not be able to screen for a TBI within 5 minutes. The latter barrier could likely be addressed through education. There are short screening tool assessments that can be delivered in <5 minutes (*e.g.*, HELPS tool).^33^

Lack of staff was a concern to some participants when asked about screening for TBIs. Lack of space was also a concern as some shelters do not have a quiet space in which to conduct TBI screening. While important, neither of these concerns were addressed in the intervention recommendations as it is believed unlikely that women's shelters have the resources available to immediately address these concerns by employing more staff or securing additional space.

Finally, establishing formal policies that require staff to assess for TBIs, and training staff to use a conversational assessment that occurs after intake will likely address concerns that a screening tool does not exist.

Our findings also indicate that staff are not screening for TBIs because of the influences of their clients. Staff did not want to cause additional trauma to an already-stressed survivor by asking about TBIs. Staff also discussed the importance of nurturing an alliance between themselves and the client. As such, staff said that they would not screen for TBIs if they did not first obtain informed consent. These concerns may be mitigated by providing the option to screen for TBI through an informal conversational style rather than a formal scored screening process. Applying a conversational style assessment may create a more relaxing context for the client, increasing their willingness to be screened, and adding peace of mind for staff.

Finally, we found that staff will not screen a client for a TBI in contexts, which make them feel the process is not useful, that is, if the client is intoxicated, deaf or hard of hearing, blind, illiterate, and so on. Although some of these circumstances would make it more difficult to screen for a TBI, incorporating a variety of real-world scenarios, and opportunities for practice and feedback, in training workshops may help staff feel more comfortable.

### Strengths and limitations

The recommendations presented from our research were developed with many strengths. First, this study provides a national representation of staff at women's shelters. Nine of 10 provinces were represented within the sample in phase 1. Second, this research was grounded in behavior change theory, which allowed recommendations to be developed through a systematic and evidence-based process. Third, an iKT approach was used throughout the research process.

As with all research, this study was not without its limitations. First, the TDF is a framework and not a theory. Therefore, it does not provide insight into how the domains interconnect.

Second, we were limited to collecting TDF data via a questionnaire that was not specifically developed and validated for the TBI-IPV context in phase 1. According to Huijg et al., reinforcement, goals, and behavioral regulation are unable to be discriminately measured^19^ and as such were not included in the phase 1 questionnaire.

Third, most of our participants were ethnically white. Staff with other ethnic backgrounds may experience different barriers to screening for TBIs. Future research should measure the barriers experienced by other ethnic groups as they may need different intervention recommendations.

Finally, our recommendations have not been tested. Further research is likely needed to support our findings. The intervention recommendations need to be implemented and evaluated to fully understand the effect they will have on TBI screening in women's shelters.

## Conclusions

This research is the first theory-informed study to examine how TBI screening in women's shelters to change that, a multicomponent intervention is required. It provides five intervention recommendations that were co-developed as part of a systematic and evidence-based process. The developed recommendations hold the potential to greatly impact the health of women who have experienced a TBI from IPV.

Whereas this study demonstrated that screening has the potential to positively impact the lives of women who have experienced IPV-related TBI, it is important to consider the broader context under which it would occur. For example, if appropriate TBI-informed supports are not readily available in the community, or if TBI screening leads to an increased risk of stigmatization in family court proceedings, or the survivor becomes more vulnerable and at risk of harm from her abusive partner, then the utility of such screening comes in to question. Further research is required to better understand this context and how best to ensure that the advantages of TBI screening outweigh any disadvantages.

Interventions aimed at promoting TBI screening at women's shelters should follow these recommendations to help ensure successful interventions and hopefully increase the number of TBI supports provided to women who have experienced a TBI from IPV. Ultimately, these recommendations hold the potential to help women who have experienced a TBI from IPV recover from their TBI(s), leave abusive relationships, and improve their quality of life.

## Abbreviations Used

BCW: Behavior Change Wheel
iKT: integrated knowledge translation
IPV: intimate partner violence
PABAK: prevalence-adjusted bias-adjusted kappa
TBIs: traumatic brain injuries
TDF: Theoretical Domains Framework
